# Self-Healing Behavior of Polymer/Protein Hybrid Hydrogels

**DOI:** 10.3390/polym14010130

**Published:** 2021-12-30

**Authors:** Maria Bercea

**Affiliations:** “Petru Poni” Institute of Macromolecular Chemistry, 41-A Grigore Ghica Voda Alley, 700487 Iasi, Romania; bercea@icmpp.ro

**Keywords:** self-healing, hybrid network, viscoelasticity, PVA, HPC, BSA

## Abstract

The paper presents the viscoelastic properties of new hybrid hydrogels containing poly(vinyl alcohol) (PVA), hydroxypropylcellulose (HPC), bovine serum albumin (BSA) and reduced glutathione (GSH). After heating the mixture at 55 °C, in the presence of GSH, a weak network is formed due to partial BSA unfolding. By applying three successive freezing/thawing cycles, a stable porous network structure with elastic properties is designed, as evidenced by SEM and rheology. The hydrogels exhibit self-healing properties when the samples are cut into two pieces; the intermolecular interactions are reestablished in time and therefore the fragments repair themselves. The effects of the BSA content, loaded deformation and temperature on the self-healing ability of hydrogels are presented and discussed through rheological data. Due to their versatile viscoelastic behavior, the properties of PVA/HPC/BSA hydrogels can be tuned during their preparation in order to achieve suitable biomaterials for targeted applications.

## 1. Introduction

In nature, the damage or failure of different living tissues can be spontaneously healed without the action of some external stimuli. Inspired from biological systems, this extraordinary ability was translated to smart polymer networks or other systems, and it is of high interest for various engineering applications [1,2,3]. Thus, during the recent years, many efforts were devoted to the design and investigation of materials with self-healing behavior in order to improve the required performances in using conditions or to extend their service lifetime in various applications. These smart materials are able to recover spontaneously their initial state or to repair themselves after damage or degradation. Thus, they recover the functionality and structural integrity by assuring a high rate of healing as compared with the rate of damage.

Analogous to the structures present in biological systems at different length scales, a remarkable progress was registered in developing new functional materials for regenerative medicine, pharmaceutics or electronics and advanced technologies [4,5,6,7,8,9,10,11,12]. Due to high elasticity, superabsorbence and excellent mechanical properties, synthetic hydrogels with porous structure are widely used in biomedical applications for controlled drug release, tissue engineering, implantable devices, wound dressings, contact lenses, biosensors or actuators, etc. [10,11,12,13,14]. In particular, various biomaterials can be easily obtained by environmental-friendly chemical or physical procedures applied to poly(vinyl alcohol) (PVA) [15,16], or to various combinations of PVA with other macromolecules or small molecules [17,18,19,20,21,22]. The high interest in the PVA-based hydrogels is due to the exceptional mechanical properties [4,23] and their performances in multifunctional biomaterials [19,20,21,22,23,24], but also to their self-healing properties [5,9,11] and high elasticity proved in creep and recovery tests [9,11,19].

Certain other specific functions that belong to natural tissue can be conferred by introducing natural molecules (proteins, peptides, polysaccharides, clays, etc.) in the hydrogel structure: biocompatibility, bioadhesivity, biodegradability, cell proliferation ability, antimicrobial activity, low cytotoxicity, etc. [25,26,27,28,29]. However, the hydrogels composed only from natural biomolecules [26,27] often exhibit a very long gelation time and poor mechanical properties, limiting their applications. Many new strategies to form double networks have emerged in order to improve the mechanical properties and biological compatibility of materials, such as dual physically/chemically crosslinking hydrogels [12,28,29,30,31,32,33,34,35,36]. Multifunctional hydrogels based on synthetic or natural polymers combined with proteins, peptides or active enzymes represent in most cases a synergistic approach that allows the design of materials with targeted properties in various applications. These hybrid materials present improved mechanical and structural stability, functionality and biocompatibility, and they are able to integrate and regenerate the tissue [26,27,29,37].

Serum albumins have the ability to bind various biological molecules, either hydrophobic or hydrophilic, cationic or anionic [38,39]. Bovine serum albumin (BSA) is a globular protein with a structure homologous with human serum albumin, often used as a model bioactive protein [40] or for enzyme separation and purification by specific complexation [41], but also to prepare materials for biomedical applications [42,43]. Reduced glutathione (GSH) is a tripeptide (γ-L-glutamyl-L-cysteinylglycine) well known as a natural antioxidant due to its redox properties. The majority of diseases are associated with a decrease in GSH content in the living cells, combined with various oxidative stress states. Different approaches are considered as emerging tools to increase GSH bioavailability and the potential uses in nanotechnologies, as for example the design of stimuli responsive systems for targeted drug delivery [44]. According to in vitro and in vivo investigations [45], GSH conjugated BSA nanoparticles represent a promising non-toxic brain drug delivery system. In presence of GSH, the tris-citric acid extender improves the freezability, post-thaw quality of buffalo bull spermatozoa and in vivo fertility [46]. The addition of 1 mM GSH to the freezing and thawing extenders increases the motility of the human sperm by improving the level of sulfhydryl groups on membrane proteins [47]. Recently, it was shown that GSH inhibits the dimerization and wild type SARS-Cov-2 Mpro activity [48].

PVA, in combination with BSA and hydroxypropylcellulose (HPC), in the presence of GSH, was recently used to prepare porous hydrogels by the freezing/thawing method. It was found that the obtained physical networks have high hydrophilicity and swelling capacity in solution [29]. In this paper, double protein/polymer networks are prepared. The physical hydrogels are formed by S-S interactions of unfolded BSA in the presence of GSH, and then PVA junction points resulted by applying successive freezing/thawing cycles. The entire range of the polymer/BSA composition was explored, and the self-healing ability was investigated in different conditions of deformation and temperature.

## 2. Materials and Methods

### 2.1. Materials

**Scheme 1:**
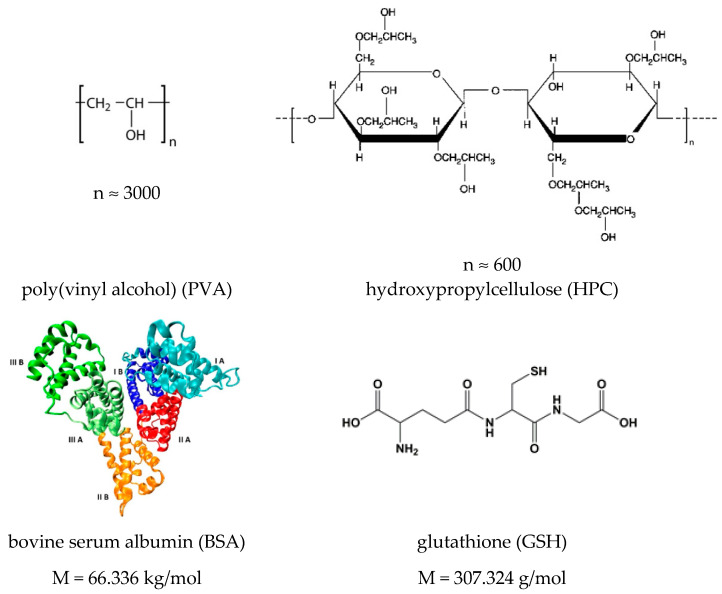
Structure of polymers, protein and tripeptide used for hydrogel preparation. The structural subdomains of BSA are displayed with different colors (Reprinted with permission from Ref. [50] copyright 2012 Elsevier Ltd.).

Poly(vinyl alcohol) (PVA, 130 kg/mol, 99% hydrolyzed), bovine serum albumin (BSA, 66.336 kg/mol, 98% purity) and reduced glutathione (GSH, 307 g/mol) were purchased from Sigma-Aldrich (Taufkirchen, Germany) and used as received. A sample of Klucel hydroxypropylcellulose (HPC) with M_w_ of 370 kg/mol was kindly offered by Ashland Aqualon [49]. The structure of all chemicals used for hydrogel preparation is given in Scheme 1.

PVA, HPC and BSA solutions of 5 g/dL were prepared in Millipore water. PVA solution was dissolved under magnetic stirring at 90 °C for approx. 4 h until the homogeneous solution was obtained; then, the sample was kept overnight at room temperature. BSA was dissolved in water by slightly shaking at room temperature (~25 °C) by means of a rolling mixer; then, the resulted homogeneous solutions were stored at 4 °C for 24 h. HPC was dissolved under magnetic stirring at room temperature and stored at 4 °C. All solutions were filtered by using a 0.45 μm Millipore filter. A stock aqueous solution of 5 g/dL concentration containing 10% HPC and 90% PVA (wt./wt.) was used for hydrogel preparation; this composition was kept constant in the present study; thus, the HPC/PVA mixture was denoted as “polymer”. This polymer solution was mixed with 5% BSA solution in appropriate weight ratios in order to achieve different weight fractions of BSA (*w*_BSA_) in the polymer/protein mixture: 0.01, 0.05, 0.1, 0.2, 0.3, 0.4, 0.5, 0.6, 0.7, 0.8, 0.9%. The content and specification of the samples are presented in Table 1. The systems were kept at room temperature for 12 h. The pH was adjusted to 7.4 by using 0.1 M solution of NaOH. 1 mmol/L GSH was added to each sample (this tripeptide content is found in the normal human cells) under continuous stirring.

### 2.2. Hydrogel Preparation

The solutions containing PVA, HPC, BSA and GSH were subjected to 3 successive freezing/thawing cycles resulting porous hydrogels. For low BSA content, 3 freezing/thawing cycles are optimum for a porous hydrogel formation; for samples with a high content of BSA, several additional cycles were applied to assure the network structure with the required characteristics [29]. In the present paper, before submitting to the freezing/thawing procedure, the polymer/protein/peptide solutions were heated at 55 °C; thus, the BSA macromolecules reached partially the disordered state [51,52], and they were able to interact each other to form a network [53]. The temperature induces protein-protein interactions [54,55], whereas HPC chains develop hydrophobic interactions [56,57,58]. In addition, GSH binds spontaneously BSA macromolecules through hydrogen bonds and van der Waals interactions (enthalpy driven process) [59]; thus, a weak network is formed by physical interactions. The samples were cooled down at room temperature and then submitted to 3 successive freezing/thawing cycles; the freezing at −20 °C for 3 h was followed by a slow thawing up to 37 °C for 21 h. PVA-PVA junctions formed during the slow thawing ensure the development of stable double networks as a result of the multiple interactions that are established between the components of the system. After freezing/thawing cycles, the crystalline aggregates formed by PVA chains acts as knots. The PVA segments from the amorphous domains and HPC long chains ensure the connectivity of the porous network.

### 2.3. Scanning Electron Microscopy Studies

The morphology of freeze-dried hydrogels was examined on a cross-section of the samples by using the Quanta 200 scanning electron microscope (SEM) with an operating voltage of 20 kV. The average size of pores was estimated directly from the SEM micrographs by using Image J Software, and an average value of at least 60 dimensions was determined.

### 2.4. Rheological Investigation

The rheological investigation was carried out with a MCR 302 Anton-Paar rheometer equipped with plane-plane geometry (the diameter of the upper plate of 50 mm, gap of 500 μm) and a Peltier device for a rigorous temperature control. An anti-evaporation device was used to create a saturated atmosphere of water in the neighborhood of the sample, preventing its evaporation.

The present investigation is focused on the viscoelastic characteristics of hybrid hydrogels and how to achieve, from rheological measurements, information on network performances in conditions similar to their use in different applications. Thus, the dynamic behavior of hydrogels with various polymer/BSA compositions was investigated at 37 °C in oscillatory shear conditions, in the linear range of viscoelasticity which was determined for each sample in an amplitude sweep test. The viscoelastic moduli (G′ and G″) were determined in frequency sweep tests for oscillation frequency (ω) between 0.1 rad/s and 100 rad/s (the strain, γ, of 1%). The elastic modulus (G′), as a measure of the stored energy of deformation, and the viscous modulus (G″), quantifying the dissipated energy during one cycle of deformation, were measured. The loss tangent (tan*δ*) is given by the G″/G′ ratio and provides information on the samples’ viscoelasticity.

Self-healing behavior was investigated in oscillatory shear experiments carried out at different temperatures and strain values. The viscoelastic moduli, G’ and G”, were measured as a function of time, when ω was set constant at 1 rad/s, and γ was successively settled at low (1%), high (from 100 to 1500) and again low (1%) strain amplitude values, respectively.

## 3. Results

### 3.1. Influence of Polymer/Protein Composition on the Viscoelastic Properties of the Network

**Figure 1:**
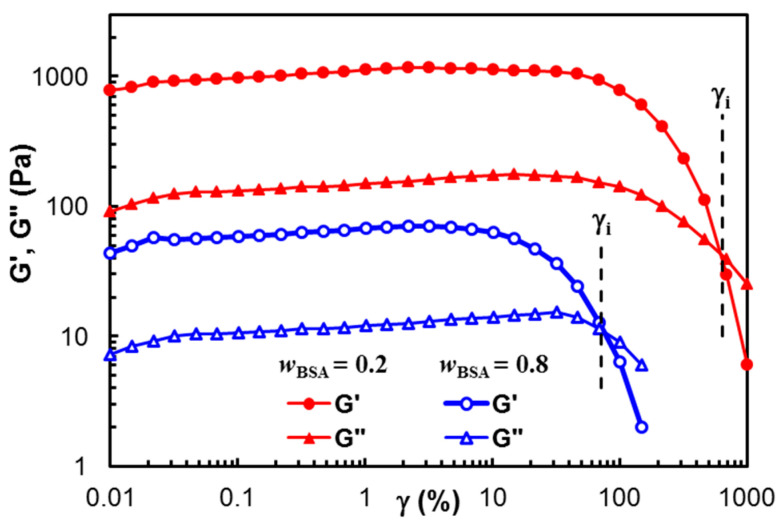
Viscoelastic moduli in amplitude sweep tests for two samples of *w*_BSA_ = 0.2 and *w*_BSA_ = 0.8.

Figure 1 presents the dependence of the viscoelastic moduli, G′ and G″, on the strain amplitude for two samples of different polymer/BSA compositions: *w*_BSA_ = 0.2 and *w*_BSA_ = 0.8. A gel-like structure is observed for both samples (G′ > G″), and the linear range of viscoelasticity is more extended for high polymer content. The value of γ_i_ (the strain at which G′ = G″) decreases with an increasing BSA content (Table 1). Generally, the hard network structures (which are usually obtained by chemical crosslinking) present a large linear viscoelastic region. In this study, the physical interactions stabilize the network at rest and in conditions of small applied strains. The linear viscoelastic range becomes narrower as the BSA content increases. This suggests that the crystalline PVA structure formed after the samples were submitted to freezing/thawing cycles contributes to the width of the linear viscoelastic domain, acting as knots of the physical network [60].

**Figure 2:**
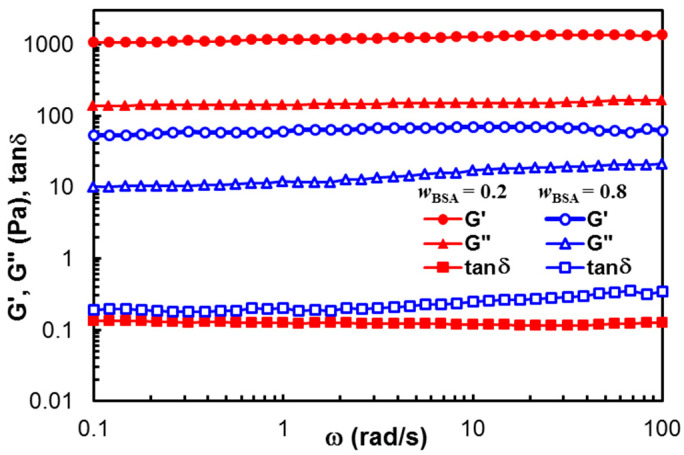
Viscoelastic parameters as a function of oscillation frequency, for hybrid hydrogels with *w*_BSA_ = 0.2 (full red symbol) and *w*_BSA_ = 0.8 (empty blue symbol) at 37 °C.

Figure 2 displays the viscoelastic moduli (G′ and G″) and the loss tangent (tan*δ*) obtained in frequency sweep tests, for the samples of *w*_BSA_ = 0.2 and *w*_BSA_ = 0.8. G’ exceeds G″ for each sample, and their values are independent on ω. The stable network structure is registered in both cases, but the polymer content influences the hydrogel strength (G’ being one decade higher for the sample with *w*_BSA_ = 0.2, as compared with those of *w*_BSA_ = 0.8). For *w*_BSA_ = 0.8, a slight increase in G″ with increasing *ω* is observed in Figure 2. Similar behavior was detected for *w*_BSA_ = 0.9. From a rheological point of view, a small value of the slope for the G″ *vs*. ω dependence (tan*δ* < 1) suggests the existence of a weak network.

**Figure 3:**
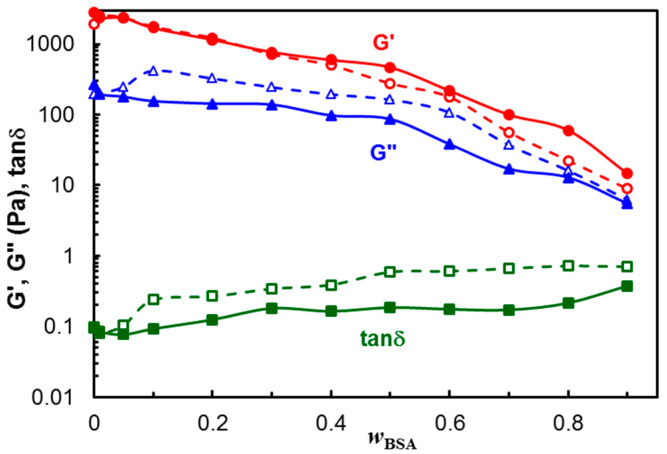
Viscoelastic parameters as a function of *w*_BSA_ for PVA/HPC and hybrid polymer/BSA hydrogels after 3 freezing thawing cycles, ω = 1 rad/s, γ = 1%, at 25 °C (empty symbol, dashed line) and 37 °C (full symbol, continuous line). The lines are a guide to the eye.

The dependences of G′, G″ and tan*δ* on the polymer/protein composition are given in Figure 3 (the values at *ω* = 1 rad/s were considered). The viscoelastic moduli slowly decrease with increasing BSA content for *w*_BSA_ < 0.5, and their diminution is more pronounced for *w*_BSA_ > 0.5, suggesting that polymer-polymer interactions contribute to the hydrogel strength. A synergistic effect was achieved by combining the polymer mixture with different amounts of BSA. Even the protein addition weakens the hydrogel moduli; the loss tangent, expressing the degree of viscoelasticity of the sample, remains nearly constant over a large range of the composition, especially at 37 °C.

In addition, gelation of BSA was observed at 37 °C in the presence of GSH [61] involving a reduction in intramolecular disulfide bonds (cleaved by the thiol) and crosslinking through intermolecular disulfide bonds of the resulted free sulfhydryls from the protein. This process is dependent on the pH and concentration, and it is sensitive on species of anions in the environment. In the pH range from 5 to 7, disulfide bonds of BSA are not accessible to reducing agents, but they are gradually available as the pH increases between 7 and 8. At pH = 7.4, a partial transition from the normal conformation to a basic form of BSA occurs, and some disulfide bonds are involved in the gelation process.

### 3.2. Morphology of the Hydrogels

**Figure 4:**
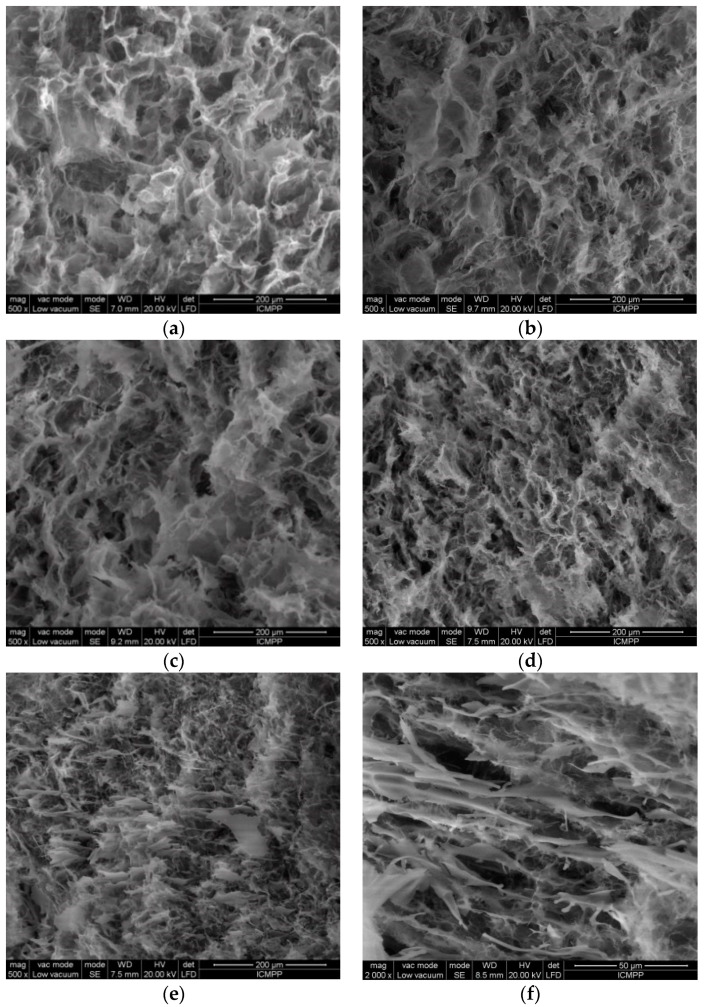
SEM micrographs of lyophilized hydrogels with different *w*_BSA_: (**a**) 0; (**b**) 0.1; (**c**) 0.2; (**d**) 0.5; (**e**) 0.8; (**f**) 0.9.

The morphology of freeze-dried hydrogels was examined by SEM using fresh prepared samples frozen in liquid nitrogen and lyophilized for 3 days. Hydrogels with high polymer content (Figure 4a–d) present a porous structure with interconnected pores. The increase in BSA content into the polymer matrix reduces the average pore size from 22 μm observed for the pure polymer hydrogel in the absence of BSA to 9.5 μm for *w*_BSA_ = 0.5.

When BSA is predominant in the hydrogel, its macromolecules are associated in clusters and aggregates; the porous structure is perturbed, and a soft hydrogel with BSA rich zones is formed (Figure 4e,f). The cleavage and rearrangement of disulfide bonds occurring in BSA are triggered by GSH presence and gentle thermal treatment before applying the freezing/thawing cycles, resulting in a disulfide bridge network. A high number of freezing/thawing cycles determines the size increase of ice-induced partial unfolding protein aggregates [29,62]; in order to avoid a strong BSA aggregation, only 3 freezing/thawing cycles were applied to all systems, and the freezing time was reduced to 3 h as compared with a previous investigation [29]. In an aqueous environment, intermolecular interactions are developed between OH groups of PVA and HPC. By applying successive freezing/thawing cycles to PVA/HPC solution, crystalline domains are formed acting as junction points, whereas polymer segments from the amorphous domains ensure the connectivity of the pores in the network. In the presence of unfolded BSA macromolecules which interact with each other after the thermal treatment, the walls of the pores are connected by physical polymer/protein interactions, and their size decreases.

### 3.3. Self-Healing Behavior as a Function of Hydrogel Composition

**Figure 5:**
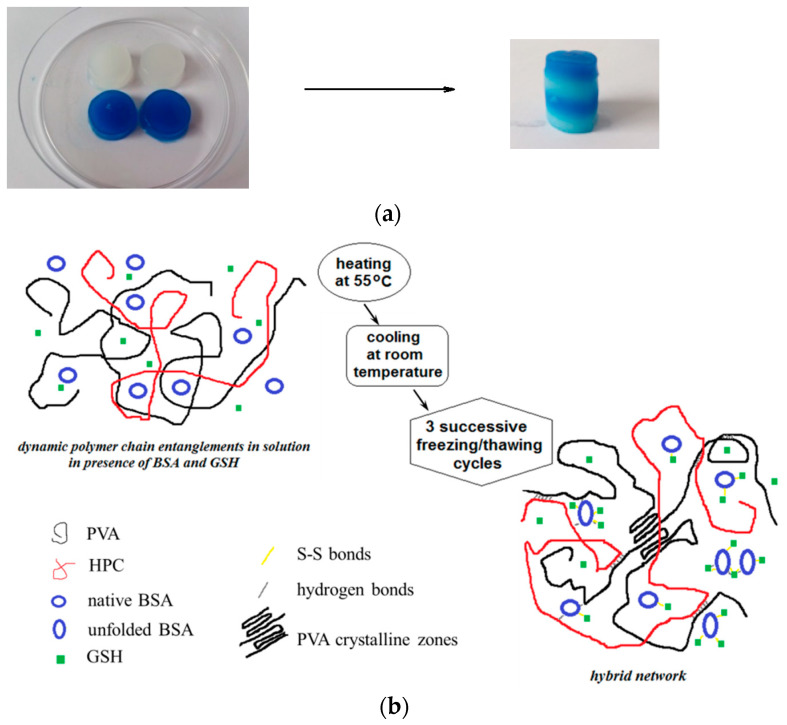
(**a**) Macroscopic image showing the self-healing behavior of hybrid hydrogels (*w*_BSA_ = 0.5). (**b**) Schematic presentation of hybrid hydrogel formation.

The self-healing ability of biomaterials is important, as it determines the extension of hydrogels’ lifespan during their applications. The hybrid hydrogels revealed an excellent self-healing ability due to multiple physical interactions developed in the multicomponent system. A rapid self-healing answer of these samples was observed macroscopically when two pieces of hydrogel were tightly welded to each other, and then they were left again in contact (Figure 5a). The hydrogel sample was cut into two pieces, and then the fragments were put together to let themselves repair. After about 3 h, the two fragments freely healed without any external stimulus. When the two hydrogel pieces were put in contact, the interactions were reestablished to a high extent. Among the different hydrogels, it was observed visually that the samples with *w*_BSA_ between 0.3 and 0.7 repaired their structure faster than the other samples. This suggests that the self-healing ability of the hybrid hydrogels is favored by the multiple interactions occurring in the investigated system (schematically shown in Figure 5b), the overall behavior being a synergistic effect of all components.

**Figure 6:**
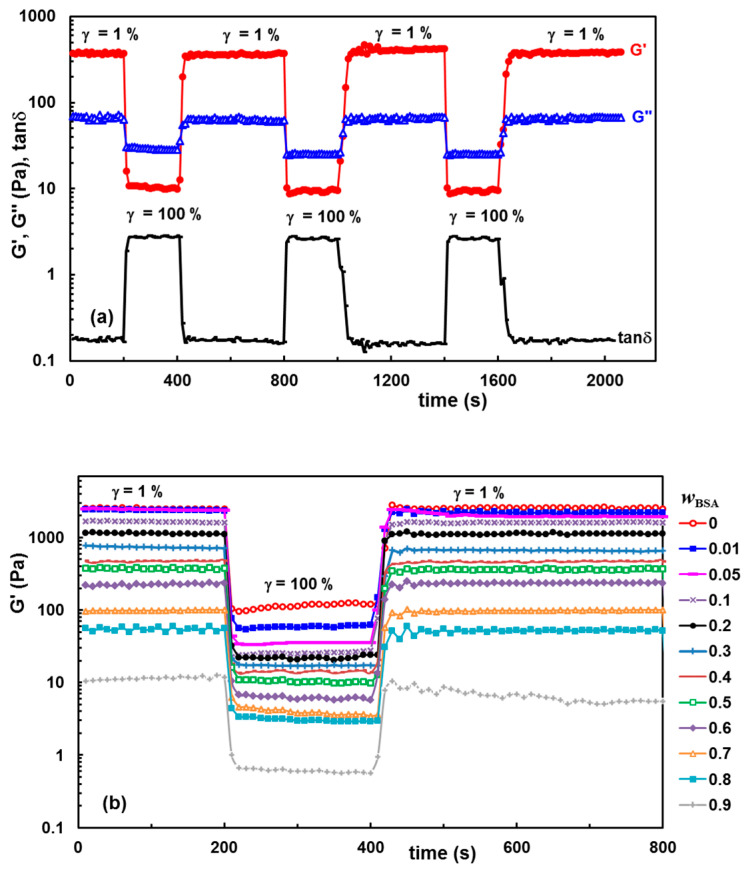
Self-healing behavior of hydrogels illustrated through consecutive step strain measurements at 37 °C: (**a**) G′, G″ and tan*δ* for the hydrogel with *w*_BSA_ = 0.5 during several successive runs of low (1%) and high (100%) strains; (**b**) G′ for different polymer/protein compositions during the first run of strain (1%–↑ 100%–↓ 1%).

The self-healing ability of the hydrogels was confirmed by dynamic rheological analysis, by analyzing the thixotropy of the samples in oscillatory shear conditions. Figure 6a reveals as an example the changes in viscoelastic parameters of hydrogel with *w*_BSA_ = 0.5 which was submitted alternatively to several cycles of low (1%) and high (100%) step strains. Figure 6b presents the data obtained for G′ using samples with a different BSA content which were submitted to alternatively 1%–100%–1% step strains.

By applying a low strain of 1% (when the rest structure is not perturbed), G′ is larger than G″, indicating the solid-like nature of the hydrogel that preserves the network integrity (Figure 6a). Then, by increasing the strain to 100%, both G′ and G″ moduli dramatically decrease, and the reached G′ value becomes lower as compared with G″, indicating that the system has a liquid-like behavior due to the temporary disruption of some physical interactions. By reducing the strain to 1%, both viscoelastic moduli return almost to the initial values reached by the sample during the first step of γ = 1%. The results obtained for the samples with *w*_BSA_ ≤ 0.8 (Figure 6b) confirm the high ability of the hydrogels to recover their rest structure after the action of the external forces, due to the reestablishment of the intermolecular interactions in time. Over a large range of composition (*w*_BSA_ ≤ 0.8), it was experimentally observed that the polymer/protein hydrogels behave very well, even after several runs of 1%–100% step strains (as exemplified in Figure 6a for the hydrogel with *w*_BSA_ = 0.5), without a significant weakening in network strength which is influenced by the hydrogel composition.

**Figure 7:**
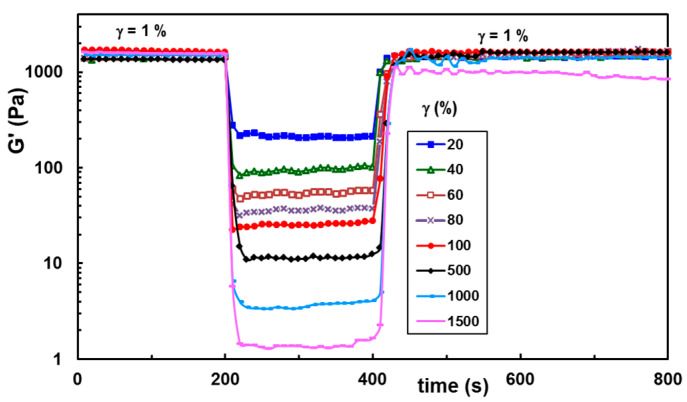
Self-healing ability of hybrid hydrogels for increasing strain values illustrated for the sample with *w*_BSA_ of 0.5.

**Figure 8:**
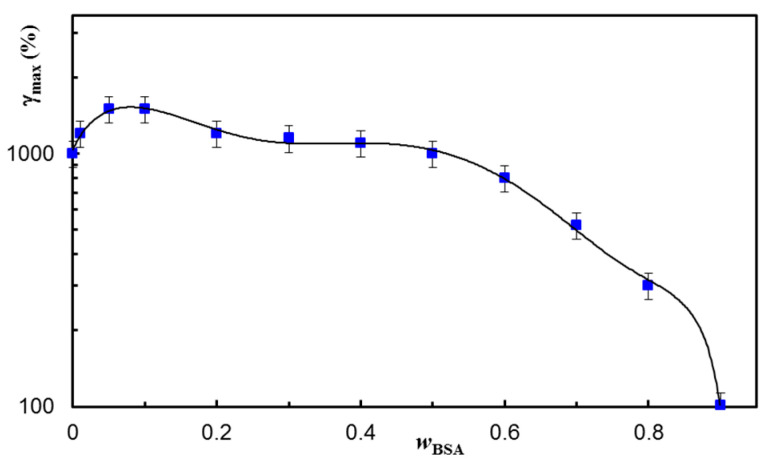
The maximum value supported by hydrogels of different compositions before the failure of their structure. The continuous line is a guide to the eye.

The self-healing ability was also tested at increasing strain values applied in the second step of the thixotropy test (Figure 7), and the results were satisfactory for a large range of applied strains. The maximum strain at which the network structure is preserved depends on the BSA content in the network (Figure 8). A synergistic behavior is observed for a small content of BSA (*w*_BSA_ in the range of 0.05 to 0.1), when the structure is recovered in time for γ ≤ 1500%.

### 3.4. Temperature Influence on the Self-Healing Ability of Hybrid Hydrogels

**Figure 9:**
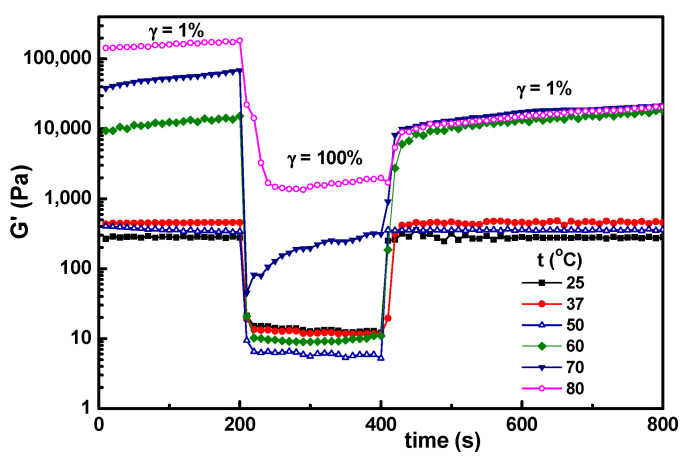
Temperature influence on the self-healing behavior of hybrid hydrogels for the sample with *w*_BSA_ = 0.5 submitted to successive step strains of 1%–100%–1%.

**Figure 10:**
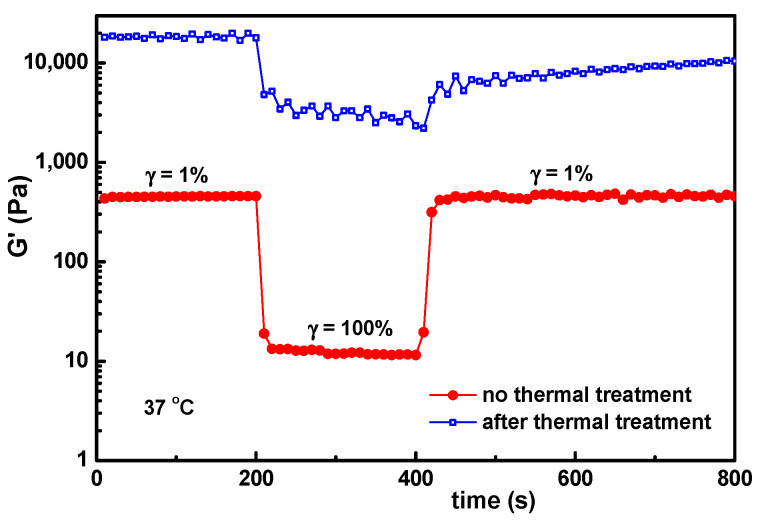
The influence of the thermal treatment (sample kept at 70 °C for 1 h and then cooled to 37 °C and thermostated for 1 h) on the self-healing behavior of the hydrogel with *w*_BSA_ = 0.5.

In the present investigation, the main interest was to conserve the structural integrity of the hybrid hydrogels during their storage and use. After 3 months, a very good stability was observed for samples with *w*_BSA_ ≤ 0.7 kept at room temperature. For a higher content of BSA, the mass loss increased in time. The effect of temperature was tested for the hydrogel sample with *w*_BSA_ of 0.5 submitted to successive step strains of 1%–100%–1% (Figure 9). The structure is recovered almost completely for the temperature range from 25 °C to 50 °C, which covers the range of interest for biomedical applications. At higher temperatures (above 60 °C), there is an important increase in G′ values, and the self-healing ability is lost, even by decreasing the temperature to 37 °C (Figure 10). The PVA-PVA interactions become weaker, and at 80 °C they vanish. The protein being denatured, the sample structure is mainly controlled by the BSA-BSA or BSA-GSH disulfide bridges and HPC hydrophobic interactions. After applying a step strain of 100%, the sample was not able to recover the rest structure. It can be observed that the structural integrity and functionality are preserved only below 50 °C. For such hybrid materials, high temperatures must be avoided if the self-healing property is required. If the sample is heated above 70 °C, the network formation by the unfolding BSA macromolecules advances further due to the association phenomena, but the intermolecular β-sheet development is irreversible [39].

BSA contains 582 amino acid residues with 17 disulfide bonds and one free thiol group at residue Cysteine (Cys)-34; approximately 17% of the amino acid residues are basic or acidic, and 28% are hydrophilic [63]. The secondary structure of native BSA is composed of 50–67% α-helix, 10% turn and about 23% random coil (no β-sheet) [39]. Previously, Peter [64] reported the presence of 15–18% β-sheet in the native protein. The shape of BSA is ellipsoid and can be separated into three domains (I, II and III, Scheme 1), each of them being stabilized by a network of disulfide bonds [39]. The multidomain structure of BSA is responsible for the irregular behavior of the protein under denaturation conditions [65].

Above 40 °C, pH, temperature and different denaturants (such as fatty acids, urea, surfactants, salts, etc.) change the secondary and tertiary structure of BSA. Temperature domain is very important for BSA structure: reversible conformational changes occur between 42 °C and 50 °C; up to 60 °C irreversible unfolding of the α-helix takes place; β-aggregation starts above 60 °C, whereas above 70 °C, the unfolding BSA advances, and a gel-like structure is developed [39,52]. The denaturation temperature is influenced by experimental conditions and the used method. As, for example, in DSC studies, the onset of denaturation for 2% BSA solutions in 0.1 M NaCl is observed at 51.9 °C, whereas the complete denaturation occurs around 64 °C [52]. After the unfolding process occurring at high temperatures, the thermal denaturation process is irreversible regardless of pH, ionic strength or additive content, due to chemical modifications, such as oxidation, deamidation, etc. [38,39,66]. The real-time dynamics throughout the denaturation process were monitored using quasi-elastic neutron scattering. In salted solutions, the protein dynamics slowed down during denaturation at high temperatures when a fractal network was formed, and this structure was maintained after cooling to room temperature [67]. The partial unfolding BSA molecules undergo thermally induced gelation, leading to stranded 3-D networks due to the exposure to the heat of the hidden hydrophobic groups from the core [53]. The changes in protein-protein interactions and in the multilevel BSA structure (at the secondary, tertiary and quaternary levels) upon heating were investigated by using various experimental techniques [66]. It was shown that up to 60 °C, the size of BSA is relatively stable, and above 65 °C, the irreversible aggregation occurs. The schematic representation from Figure 7 of the reference [66] underlines the complexity of thermally induced structural changes in BSA macromolecules.

Different processes occur during BSA isolation, storage and heating, such as S-S bond formation and reshuffling [68]. Heat induces changes in BSA conformation (protein unfolding) and inter- and intramolecular interactions of (Cys)2 residues in domains IA, IIA and IIIA, involving thiol-disulfide interchanges and thiol oxidation reactions, (Cys)2 residues playing a key role in BSA crosslinking. The disulfide bond formation and reshuffling under physiological conditions play an important role during processing different pharmaceutical and food biomaterials.

The mechanical and structural properties of thermally formed BSA hydrogels can be handled by the heating temperature and the BSA concentration. A moderate denaturation of the protein, as in the present investigation, allows a repetitive self-healing hydrogel after damage when moderate heat is induced. In our case, the viscoelastic properties of hydrogels are fully recovered due to cumulative contributions of polymers and protein. When heat is applied to the protein sample, it raises the kinetic energy and molecular vibration. Consequently, hydrogen bonds and non-polar hydrophobic interactions are interrupted, leading to protein denaturation, but disulphide bridges maintain the aggregates and even network structure at rest. In the presence of HPC and PVA long chains, the network continuity is ensured by the polymers. The PVA/PVA interactions are also destroyed at high temperatures, and the crystalline zones formed by freezing/thawing vanish. The hydrophobic interactions of HPC increase and contribute to the network structure, but they are not able to recover completely after a high deformation. Such materials are very efficient for applications involving temperatures lower than 50 °C. At higher temperatures, the protein aggregation prevents the manifestation of the self-healing ability, and, on the other hand, the crystalline zones formed by PVA chains by freezing/thawing are progressively melted. The polymer/protein composition can be optimized as a function of the conditions imposed by the application.

The strategies for achieving the self-healing characteristics and formulating smart materials for diverse applications are continuously developed; some challenges and future outlooks were discussed recently in comprehensive reviews [14,27,42,43,69,70,71,72,73]. The present paper joins the recent efforts undertaken to design self-healing hydrogels and hybrid materials [6,7,14,27,29,30,31,37,53,74] with unique and targeted properties, based on synergism, by using accessible methods, without altering the functionality and structural integrity of the components.

## 4. Conclusions

The paper presents the viscoelastic properties of self-healing hybrid hydrogels prepared by physical interactions between poly(vinyl alcohol) (PVA), hydroxypropylcellulose (HPC), bovine serum albumin (BSA) and reduced glutathione (GSH). After heating the mixture at 55 °C, a weak network is formed due to partial BSA unfolding and HPC hydrophobic interactions. By applying three successive freezing/thawing cycles, a porous structure is developed, as evidenced by SEM. The viscoelastic behavior was investigated in order to evidence the self-healing ability as a function of hydrogel composition, applied strain or temperature. The viscoelastic properties of the healed polymer/protein hydrogels were recovered to almost 100% of the original values if the strain and temperature are below a critical value. The thixotropic behavior is influenced by the BSA content, loaded deformation and temperature, and it is attributed to multiple interactions developed in the system: hydrogen bonding, hydrophobic interactions, S-S bonds, PVA/PVA interactions in the crystalline zones.

Due to their versatile viscoelastic behavior, the properties of PVA/HPC/BSA hydrogels can be tuned during their preparation in order to achieve suitable biomaterials for targeted applications. Hydrogels with moderate and low BSA content are appropriate for wound dressings and tissue engineering applications. From a rheological point of view, high BSA content formulations are suitable as injectable or targeted drug delivery biomaterials; also, they can be used for bio-printing or electrospinning at temperatures lower than 50 °C.

## Figures and Tables

**Table 1:** Sample specifications and composition *, γ_i_ (the strain at which G′ = G″ in amplitude sweep test) and tan*δ* (at 37 °C, 1 rad/s).

*w*_BSA_	% wt. Polymer **	% wt. BSA	γ_i_ (%)	tan*δ*
0	100	0	685	0.094
0.01	99	1	745	0.084
0.05	95	5	705	0.077
0.1	90	10	667	0.092
0.2	80	20	640	0.125
0.3	70	30	562	0.180
0.4	60	40	472	0.164
0.5	50	50	402	0.185
0.6	40	60	260	0.175
0.7	30	70	152	0.171
0.8	20	80	72	0.215
0.9	10	90	31	0.376

* The initial solutions contain 5 g/dL polymer, 5 g/dL BSA and 1 mmol/L GSH; ** Polymer = mixture of 10% HPC and 90% PVA (wt./wt.).

## Data Availability

Data are available on request.
